# Stigma about mental disease in Portuguese medical students: a cross-sectional study

**DOI:** 10.1186/s12909-021-02714-8

**Published:** 2021-05-10

**Authors:** Ana-Raquel Moreira, Maria-Joao Oura, Paulo Santos

**Affiliations:** 1 Department of Medicine of Community, Information and Health Decision Sciences (MEDCIDS), Faculty of Medicine, University of Porto, Porto, Portugal; 2 Center for Health Technology and Services Research (CINTESIS), Faculty of Medicine, University of Porto, Alameda Hernani Monteiro, 4200-319 Porto, Portugal

**Keywords:** Social stigma, Medical education (undergraduate), Medical schools, Mental disorders, Empathy

## Abstract

**Background:**

The stigma about mental diseases is common in the population and also in medical students, where it may condition their future practice and the way they deal with these patients.

**Aim:**

To evaluate and characterize the stigma on mental diseases in Portuguese sixth-year medical students, based on a clinical scenario of a classmate suffering from a mental disorder.

**Methods:**

Observational cross-sectional study, involving sixth-year students of all Portuguese medical schools. We applied an online self-response questionnaire, using the Portuguese version of the Attribution Questionnaire AQ-9, and a vignette of a classmate colleague, presenting mental illness symptoms. Stigma scores were calculated. We used logistic regression to estimate the effect of social determinants on stigma pattern, and we analysed the correlation between 9 variables evaluated by the AQ-9 and total stigma.

**Results:**

A total of 501 participants were included for analysis (69.5% females, median age of 24 years old). Medical students were available to help in the proposed clinical scenario (6.93/9.00; 95%CI:6.77–7.10), if necessary using coercion for treatment (3.85; 95%CI:3.63–4.07), because they felt pity (6.86; 95%CI:6.67–7.06) and they perceived some kind of dangerousness (4.06; 95%CI:3.84–4.28). Stigma was lower in students having a personal history of mental illness (OR:0.498; 95%CI:0.324–0.767; *p* = 0.002) and in those with positive familial history (OR: 0.691; 95%CI:0.485–0.986; *p* = 0.041).

**Conclusion:**

Our results show the importance of implementing anti-stigma education, to improve medical students’ attitudes towards peers living with mental diseases.

**Supplementary Information:**

The online version contains supplementary material available at 10.1186/s12909-021-02714-8.

## Introduction

Mental disorders are often subject of social stigmatisation and discrimination. The stigma can be defined in many ways, since it was first introduced in 1961 by Erving Goffman. The stigma is “an attribute considered undesirable and unpleasant by society and which differentiates the stigmatized person from other members of the community that he or she should belong to” [1] that can ultimately lead to the exclusion in many critical life domains [2].

Although mental illness is common worldwide, negative attitudes towards people living with mental disorders endure. In addition to Schomerus [3], Pescosolido et al. recently found that despite an increase in knowledge about mental illness in the past 20 years, attitudes towards patients have not improved [4]. Stigmatizing behaviours continue to be reported in several scenarios, mostly in the community but also in the workplace and healthcare services [5]. Like the general population, medical students may have stereotypical views about people with mental diseases, thinking these people are unlikely to recover and can be dangerous. Although these negative attitudes could exist before medical school, some students may also be contaminated and influenced by negative attitudes towards psychiatry and psychiatric patients from their tutors [6, 7]. The professional deontological commitment can hide the problem in the verbalization of more inclusive expressions, but that eventually remain only in the intentions, not in the acts. Thus, it is interesting to situate the problem in terms of the personal life aspects where reason and emotion are mixed and less prone to an academically correct answer.

Moreover, medical students are a population under a lot of physical and emotional stress. In fact, mental health diseases such as severe anxiety, burnout and depression are more common in this population compared to the age-matched general population; they also have a lower quality of life [8–10]. Rotenstein found a worldwide prevalence of 27.2% for depression, with 11.1% for suicidal ideation, in medical students, and 28.8% in residents, suggesting that depression affects all levels of medical training [11]. Depressive symptoms poorly affect the long-term health of physicians, making necessary to establish measures of prevention and support for those at risk. Several factors are related to the high prevalence of mental health disorders in medical students. The academic election may favour specific profiles on students such as perfectionism, performance-based self-esteem and a hand, medical students show higher levels of sleep deprivation, competitiveness, lack of time for social activities and regular physical activity and insufficient support systems [12].

However, students, as doctors, are supposed to be strong. This can lead to self-stigma, defined as the incorporation of other people’s stereotypes about those with mental health illness into beliefs about oneself [13]. Adopting social stigma leads to lose confidence, constituting an obstacle in performing social and professional roles [14]. Givens et al. reported that depressed medical students have more often stigmatizing behaviours towards psychiatric patients than non-depressed medical students [15]. This fear of exposure to stigmatisation can be a tremendous obstacle in seeking treatment.

Depressed doctors and medical students receive the appropriate treatment less often than general people with depression, despite, in theory, they have better access to healthcare. Barriers to look for help include several aspects such as lack of time, lack of privacy, stigma about psychiatric treatment, financial difficulties and potential negative effects on their professional career [14, 16]. Ultimately, students may believe that it is preferable to live with these difficulties alone instead of seeking professional help.

This vicious cycle of stigma and self-stigma can lead both medical students and doctors to live with untreated depression and its numerous consequences: poor academic performance, low productivity, higher levels of school dropout and burnout [15]. Moreover, they may be a font of discrimination among psychiatric patients, which ultimately leads to premature death in people with mental illness [17, 18].

Medical students are an important group to target amongst healthcare providers regarding attitudes towards mentally ill people, since they are the doctors of tomorrow and will be responsible for shaping medical culture [19]. It is crucial to establish an efficient anti-stigma education program to prevent the consequences of this stigmatizing behaviour on mental disorders in future doctors. In Portugal, the medical courses are approved by the bologna process and take 6 years to complete, with a first pre-clinical part (3 years), a clinical part (2 years) and a final year of professional training. In the last year, we expect students to have a posture close to a resident with a progressive gain of independence in their performance, both in technical tasks and clinical reasoning as in ethical and deontological skills.

Our study aims to evaluate and to characterize the stigma about mental diseases in Portuguese sixth-year medical students. Specifically, we are interested in understanding stigma toward a peer experiencing a mental health crisis and prospect for the determinants able to predict the stigmatizing attitudes.

## Methods

We conducted an observational cross-sectional study, involving sixth-year students of all Portuguese medical schools. We applied an online self-response questionnaire, using the preliminary version of the Portuguese version of Attribution Questionnaire AQ-9 to characterize de stigma.

In Portugal, there are about 1200 students attending the sixth-year of medical school, in eight medical faculties: Faculdade de Medicina da Universidade do Porto (FMUP), Instituto de Ciências Biomédicas Abel Salazar (ICBAS), Faculdade de Medicina da Universidade de Lisboa, (FML) NOVA Medical School Lisboa, Escola de Medicina da Universidade do Minho (EM), Faculdade de Medicina da Universidade de Coimbra (FMUC) Faculdade de Ciências da Saúde da Universidade da Beira Interior (UBI) and Universidade do Algarve (UAlg). All students were eligible for participation.

The data collection occurred from September 2019 to the end of January 2020. All eligible students were invited to participate in the survey on four different occasions through the institutional e-mail system.

The self-response questionnaire consisted of three parts: (1) measure of stigma, using the Portuguese version of the Attribution Questionnaire AQ-9, developed by Corrigan [20, 21]; (2) perceived medical school stress using the 10 items Perceived Stress Scale (PSS) [22]; and (3) social and demographic variables: age, gender, enough sleeping hours, eating habits, spirituality, time management, social life satisfaction, academic life satisfaction, the presence of financial difficulties, family support, the place of residence (home/away from home), the frequency of home visits (if applicable), the worries about the future, history of mental illness in the present or the past, and the family history of mental illness.

The Attribution Questionnaire AQ-9 measures stigma by evaluating 9 factors: pity, anger, danger, fear, responsibility, help, segregation, avoidance and coercion, representing nine stereotypes about mental disease. This scale starts with a case report of a medical student suffering from burnout, followed by 9 assertions, one for each dimension, answered by a Likert scale of 1 (no or nothing) to 9 (very or completely). The global score points from 9 to 81 points: the higher the score, the great the stigma [23].

The Perceived Stress Scale (PSS) measured the stress among students. This 10 items’ scale was developed by Cohen et al. [24] and translated to the Portuguese language by Pais-Ribeiro and Marques [22]. It classifies how often participants have found their lives unpredictable, uncontrollable and overloaded in the last month in a continuous score ranging from 0 to 40, with higher punctuation meaning higher stress. It has an internal consistency (Cronbach’s α) of 0.87 and an item total correlation ranging from 0.32 to 0.82, with the majority above 0.60. In the Portuguese validation, a dichotomic categorization by the 80th percentile was assumed (total score of 22 in the females and 20 in the males), above which stress was classified as pathological.

The story we proposed for appraisal was “*José* is a 23-year-old medical student who is studying for National Test for access to the specialization. Recently, he has slept three hours per night, cries constantly, feels exhausted and is not motivated to study. He isolated himself from his friends and showed some aggression towards his parents. He has been hospitalized twice for episodes of self-harm.”

Other works show that asking participants to answer to a specific person with mental disease, rather than people with mental diseases in general, leads to a more sensitive measure of attitudes that better corresponds to concurrent validators [25]. The result produces a representative score of each stereotype and the stigma is directly proportional to the score value. The higher the score, the greatest the stigma; test–retest and confirmatory factor analysis verified the reliability and validity of this model [26].

The study protocol was assessed and approved by the Ethical Committee of *Hospital de S. Joao - Faculdade de Medicina da Universidade do Porto*. We strictly followed the principles of the Helsinki Declaration and the Oviedo Convention about the protection of human rights in the biomedical investigation. The first page of the web-form, before the questionnaire itself, included information for participants, and asked for their explicit written consent, allowing the refusal, which led to automatically drop out of the study.

We used descriptive and inferential statistics. The normality of distribution was checked by Kolmogorov-Smirnov test. Stigma did not present normal distribution and we opted for its dichotomization around the median in high and low, and the utilization of the logistic regression to estimate the effect of the determinants. Confidence intervals were calculated through the modified Wald method. Pearson correlation was used to analyse the weight of each dimension in the total stigma. The significance level was set at 0.05. Data were encoded and registered in a Microsoft Office Excel 2013VR database and analysed using IBM SPSS Statistics VR, version 25.0 (IBM Corp., Armonk, NY, USA®).

## Results

A total of 501 participants out of 559 were considered for analysis (89.6%). Fifty-eight questionnaires were invalid because students did not satisfy the inclusion criteria. As expected by the demography of medical students in Portugal, females were the main group (69.5%) and the median age was 24 (±2.5). Also, as expected by the distribution of students across national colleges, FMUP was the most represented college (29.7%), followed by FMUC and FML (19.6 and 13.2%, respectively). The other faculties had a smaller representation: 11.4% of answers from UM, 6.0% from NOVA and 3.2% from UALg. Table 1 shows the demographic characteristics of our sample.

**Table 1:** Sociodemographic characteristics of medical students

Characteristics	N (%)
Gender	
Male	153 (30.5%)
Female	348 (69.5%)
Mean age in years (SD)	24.0 (±2.5)
Medical school	
FMUP	149 (29.7%)
ICBAS	57 (11.4%)
UM	37 (7.4%)
UBI	48 (9.6%)
FMUC	98 (19.6%)
FMUL	66 (13.2%)
NOVA	30 (6.0%)
UAlg	16 (3.2%)
Lives away from home ^a^	319 (63.7%)
Visits home frequently (in those living away from home) ^a^	248 (78.0%)
Sleeps enough hours ^a^	248 (49.5%)
Maintains a balanced diet ^a^	370 (73.9%)
Able to manage time ^a^	283 (56.5%)
Social life satisfaction ^a^	216 (43.1%)
Academic life satisfaction ^a^	208 (41.5%)
Financial difficulties ^a^	57 (11.4%)
Family support ^a^	481 (96.0%)
Worries about the future ^a^	375 (74.9%)
Spirituality feelings ^a^	231 (46.1%)
Students presenting stress †	250 (49.9%)
New exam model perception	
Less intimidating	181 (36.1%)
Equally intimidating	121 (24.2%)
More intimidating	199 (39.7%)
Had/has mental illness	17 (23.4%)
Had/has family with mental illness	283 (56.5%)

^a^ Students answering 4 or 5 in the 5 points Likert scale from 1 (nothing) to 5 (completely); † Perceived Stress Scale; *FMUC - Faculdade de Medicina da Universidade de Coimbra*; *FMUL - Faculdade de Medicina da Universidade de Lisboa*; *FMUP - Faculdade de Medicina da Universidade do Porto*; *ICBAS - Instituto de Ciências Biomédicas Abel Salazar*; *NOVA - NOVA Medical School*; *SD* standard deviation; *UAlg - Universidade do Algarve*; *UBI - Faculdade de Ciências da Saúde da Universidade da Beira Interior*; *UM - Escola de Medicina da Universidade do Minho*

The AQ-9 scale assessed stigma behaviour. In the global assessment, the stigma pointed 33.6 (95%CI: 32.8–34.5), varying from 11 to 81. Table 2 shows the score of each factor. Help (6.93) and pity (6.86) were the factors most pointed, followed by danger (4.06) and coercion (3.85). The items that obtained the lowest scores were anger (1.69) and blame (2.30).

**Table 2:** Score of factors related to stigma, evaluated by AQ-9

	Mean (95%CI)
Pity	6.86 (6.67–7.06)
Danger	4.06 (3.84–4.28)
Fear	3.22 (3.01–3.42)
Blame	2.30 (2.15–2.45)
Segregation	2.34 (2.18–2.50)
Anger	1.69 (1.58–1.81)
Help	6.93 (6.77–7.10)
Avoidance	2.36 (2.22–2.51)
Coercion	3.85 (3.63–4.07)

*CI* - confidence interval

**Fig. 1:**
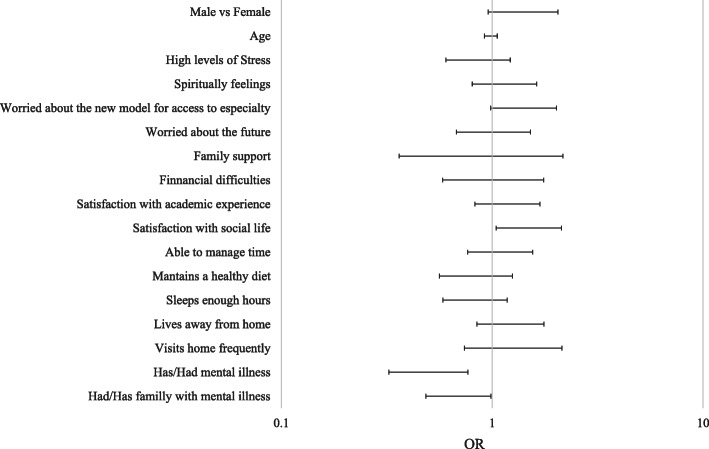
Relation between stigma and sociodemographic factors. OR: odds ratio

We checked the relationship between stigma and its determinants (Fig. 1). The stigma was higher when students presented better social life satisfaction (OR:1.493,95%CI:1.046–2.131, *p* = 0.027), and lower if they presented mental illness in the present or the past (OR:0.498, 95%CI:0.324–0.767, *p* = 0.002) or history of family mental illness (OR: 0.691, 95%CI:0.485–0.986, *p* = 0.041).

The weight of each dimension in the stigma was tested by the correlation between the total score and each factor (Table 3), defining the profile of students most associated with stigma. The higher stigma was associated with a higher perception of dangerousness (ρ = 0.729; *p* < 0.001), higher fear (ρ = 0.727; p < 0.001), and higher segregation feelings (ρ = 0.621; p < 0.001). Students who fear and are scared by a colleague with mental illness are also the ones who think that it would be best to submit the patient to treatment against his will in a psychiatric hospital.

**Table 3:** The correlation between the 9 variables evaluated by the AQ-9 and total stigma

	Score total stigma	Pity	Danger	Fear	Blame	Segregation	Anger	Help	Avoidance
**I feel pity for José**	0.406^**^	–							
**How dangerous is José?**	0.729^**^	0.153^**^	–						
**Would José scare you?**	0.727^**^	0.129^**^	0.619^**^	–					
**I think José is to blame for is illness**	0.439^**^	−0.068^**^	0.237^**^	0.305^**^	–				
**I think it would be best for José’s community if he were put away in a psychiatric hospital**	0.621^**^	0.074^**^	0.394^**^	0.347^**^	0.258^**^	–			
**How angry would you be with José?**	0.465^**^	−0.013^**^	0.249^**^	0.324^**^	0.337^**^	0.290^**^	–		
**How likely would you be to help José**	0.209^**^	0.266^**^	0.055^**^	−0.016^**^	−0.043^**^	− 0.009^**^	−0.057^**^	–	
**I would keep distance from José**	0.489^**^	0.063^**^	0.295^**^	0.339^**^	0.255^**^	0.301^**^	0.290^**^	−0.314^**^	–
**Do you think José should be forced to undergo medical treatment against his will?**	0.523^**^	0.131^**^	0.219^**^	0,225^**^	0.007	0.305^**^	0.081^**^	0.041^**^	.195^**^

** *p* < 0.01

## Discussion

Faced with a case of a mental health episode in schoolmate, our sixth-year medical students are available to help, mostly because they feel pity and, if necessary, using coercion for treatment. The items with the highest scores in the AQ-9 are help and pity. People usually feel sympathy and sorrow for those with mental health disorders. This may lead to a willingness to help but also to a different type of discrimination: that individuals with mental illness are unable to deal with adult life difficulties and need compassion by other people who can make decisions for them [27]. Reducing the stigma means that we must replace negative views with positive ones: parity and not pity [28]. Mental health patients can recover and must be empowered by health systems and by society to be independent [28].

We found an association between better social life satisfaction and higher stigmatizing attitudes. It seems that a kind of hedonistic satisfaction is associated with ignoring and devaluing others’ problems. On the other hand, the stigma was lower when students had a personal or family history of mental illness, as if they became more sensitive to mental health illness due to their own life history.

The correlation between stigma dimensions and total score shows that stigma is specially associated with feelings of fear and scare, leading to a higher likelihood of imposing a treatment even against patients’ will. Also, our results show that fear and danger were associated with a lesser chance to help the patient and to a certain level of indifference. This profile of stigma has been described before. The conceptual model described by Corrigan et al. [20] implies that negative emotional responses such as anger and fear are linked to the belief that mental illness is under persons’ control, leading to discriminatory behaviours such as segregation and coercion. According to other studies [29, 30], our results also show lesser levels of stigma in people with a history of personal or familial mental illness. Individuals who have familiar with mental health disorders are more likely to help and less likely to believe that these patients are dangerous, which leads to less social detachment [30].

Although mental health problems are common in medical students, and even higher than the age-matched population [31], access to psychiatric and psychological help remains limited. Many students fail to recognise the importance of seeking help, due to self-stigma, and others have financial and time management difficulties [32]. These constraints indicate the need to intensify the support for medical students with mental health problems, both therapeutically as preventively, and creating healthy environments, such as self-help groups and activities to promote psychological well-being. This is also a responsibility of the institutions of higher education, as World Health Organization recommends to implement the health promoting University [33].

Medical students are a very important group to target in terms of anti-stigma education. This population tend to be more amenable to influence and, as future doctors, they will be responsible for influencing future generations and possibly perpetuate the stigma towards mental health [34, 35]. Despite having better knowledge about mental health than the general population, doctors and medical students cannot distance themselves from stereotypes deeply rooted in the general population, leading to mental health stigma. This might be explained by the fact that conventional education on mental illness does not reduce stigmatizing behaviours [34]. A systematic review including 17 original studies found that despite the overtime increase of knowledge about mental illness, the stigma about depression remained constant, and the stigma about schizophrenia even got worse [36]. Several studies have shown that educational programs framing mental illness as a “brain disorder” and “biological in its origin” are associated with less blame, but with higher beliefs that people with mental illness are threatening and less likely to recover [27, 37, 38]. However, contact and education may have contradictory effects. While contact seeks to eliminate discrimination of mental illness through interactions between the general population and people in recovery, education tries to decrease stigmatizing myths of mental illness by substituting them by facts [28]. A meta-analysis including 13 randomized controlled trials showed that contact-based education is better to change the stigmatizing attitudes compared with conventional formal education. Face-to-face interactions with someone mentally ill has a greater impact than hearing a story through video. Also, follow-up effects are better for contact education [26]. In the same line, Sadow & Ryder showed that personal contact from someone with experience of mental health treatment presented a greater influence on the stigma compared with education interventions [39]. Thus, psychiatry residence during medical school may play an important role in reducing stigma towards mental health. In Portugal, every student has contact with psychiatric services, including a residence period, during 4th or 5th year. All the participants in this study had already contact with mental patients, which could induce a positive effect on the stigma.

However, the relationship between the stigma and psychiatric residence during medical education is not so clear [40, 41]. The medical community, including students, has historically negative views about mental diseases [42]. The results of different studies are heterogeneous but show that, although there is some improvement during the internships of mental health, it is not sufficiently consolidated to have an impact on stigma [43–45]. One advanced explanation is the contact of students mainly with severely ill patients in the emergency rooms and in the hospital stay, and less with outpatient consultation, where the prognosis is better and many times keeping a perfectly normal day life [46]. Moreover, it is possible that there is a lack of skills in this issue, making important to improve competences of the teachers and trainers through specific training programmes [47].

Several programmes are targeting medical students’ anti-stigma training. Some examples are the Education Not Discrimination project (END), in England [48], the Anti-stigma Intervention Curriculum project (ASIC), in Canada [35], the project “Opening minds”, in Canada [49], and its extension to 25 other countries, the responding to experienced and anticipated discrimination (READ) [50]. These programmes use the combination of various components such as knowledge, contact-based interventions and attending to the students’ internal experience of working with someone with mental problems to reduce the stigma of mental illness [35, 51]. Generally, there is a great reduction in the negative behaviour towards mental illness as comparing with common psychiatric residence, reducing fear and enhancing empathy [52, 53]. Portuguese medical students could benefit from a similar program included in the curricular structure of disciplines of psychiatry, general medicine and primary care. As far as we know, there is not any project about reducing the stigma on mental diseases in our country.

This is the first nationwide study measuring and characterizing the stigma about mental illness in medical students. Nevertheless, there are several limitations in this study that must be considered. The self-response nature of the questionnaire may lead to an information bias: students may respond according to the socially acceptable, instead of their real feelings; the optional characteristic and the distribution through the institutional e-mail may condition some selection bias, with the respondents presenting some different profile than the non-respondents. Also, the AQ-9 is widely used, but the Portuguese version is still on validation: we must be cautious about the concept of “pity”, which may have a different (more benevolent) meaning in the Portuguese language. Another question stays in the cross-sectional design It is relevant to measure the variation of the stigma during the course, to define the best time to break the cycle, and who are the best players to do it.

In conclusion, we found that the finalist medical students indicated they would be available to help a colleague with mental illness, mainly because they feel pity. The history of personal or family mental disease lessen the stigma attitudes. Students with higher stigmatizing attitudes show more feelings of danger and fear towards patients with mental disease, avoidance and a lesser chance to help those in need. It is crucial to break the cycle of stigma and self-stigma to better treat our patients and ourselves when disease comes to us, making healthcare more including and empathizing. The education system has a role in it and curricula should include special attention to the stigma as a main need in training of future doctors.

## Supplementary Information

**Additional file 1.**

## Data Availability

The datasets used and/or analysed during the current study are available from the corresponding author on reasonable request..
