# Supplementary material for: Stigma about mental disease in Portuguese medical students: a cross-sectional study
Source: BMC Med Educ. 2021 May 10;21:265. doi: 10.1186/s12909-021-02714-8 (PMC8108321; doi:10.1186/s12909-021-02714-8)
Supplement: Supplementary file 1 — Additional file 1. [file 12909_2021_2714_MOESM1_ESM.docx]

**Stigma about mental disease in Portuguese medical students: a cross-sectional study (questionnaire)**

Ana Raquel Moreira, Maria Joao Oura, Paulo Santos

1. Age:____years

2. Sex: Female/Male

3.Medical School:

1. Faculty of Medicine, University of Porto (FMUP)
2. Institute of Biomedical Sciences Abel Salazar, Porto (ICBAS)
3. School of Medicine, University of Minho (EM)
4. Faculty of Medicine, University of Coimbra (FMUC)
5. Faculty of Health Sciences, University of Beira Interior (UBI)
6. Faculty of Medicine, University of Lisboa (FML)
7. NOVA Medical School, Lisboa (NOVA)
8. Departament of biomedical sciences and medicina, University of Algarve ( DCBM-UAlg)

4. Degree

1. 1st. year
2. 2nd. year
3. 3rd. year
4. 4th. year
5. 5th. year
6. 6th. year

5. Are you displaced from your home of origin? Yes No

6. If you answered yes to the last question, do you visit your family regularly? Yes/No

7. In each of the following statements, indicate on a scale of 1 (I do not identify myself) to 5 (I identify a lot) how you currently feel about the academic year that is starting?

| I don't sleep enough time | ➀ | ➁ | ➂ | ➃ | ➄ |
| --- | --- | --- | --- | --- | --- |
| I can't make a healthy diet | ➀ | ➁ | ➂ | ➃ | ➄ |
| I think I can’t manage well my time | ➀ | ➁ | ➂ | ➃ | ➄ |
| I am satisfied with my social life | ➀ | ➁ | ➂ | ➃ | ➄ |
| I am satisfied with my student life | ➀ | ➁ | ➂ | ➃ | ➄ |
| I have financial difficulties | ➀ | ➁ | ➂ | ➃ | ➄ |
| I have no family support | ➀ | ➁ | ➂ | ➃ | ➄ |
| My future worries me | ➀ | ➁ | ➂ | ➃ | ➄ |

8. Regarding religion / spirituality I consider that:

a) There is a personal God

b) There is a kind of spirit or living force

c) I don't know what to think

d) I have doubts about the existence of a superior being or God

e) I am an atheist

9. The new model of the National Test of Access (PNA) compared to the old model:

a) It is an increased source of concern

b) The new model does not change my perception of the test

c) I think the new model is less intimidating

10. Perceived Stress Scale (Stress levels are measured using the Perceived Stress Scale, in its 10-item version translated and validated for the Portuguese population by J. Pais Ribeiro and T. Marques.) (*)

In the last month:

|  | Never | Almost Never | Sometimes | Fairly Often | Very Often |
| --- | --- | --- | --- | --- | --- |
| how often have you been upset because of something that happened unexpectedly? | 🞏 | 🞏 | 🞏 | 🞏 | 🞏 |
| how often have you felt that you were unable to control the important things in your life? | 🞏 | 🞏 | 🞏 | 🞏 | 🞏 |
| how often have you felt nervous and “stressed”? | 🞏 | 🞏 | 🞏 | 🞏 | 🞏 |
| how often have you felt confident about your ability to handle your personal problems? | 🞏 | 🞏 | 🞏 | 🞏 | 🞏 |
| how often have you felt that things were going your way? | 🞏 | 🞏 | 🞏 | 🞏 | 🞏 |
| how often have you found that you could not cope with all the things that you had to do? | 🞏 | 🞏 | 🞏 | 🞏 | 🞏 |
| how often have you been able to control irritations in your life? | 🞏 | 🞏 | 🞏 | 🞏 | 🞏 |
| how often have you felt that you were on top of things? | 🞏 | 🞏 | 🞏 | 🞏 | 🞏 |
| how often have you been angered because of things that were outside of your control? | 🞏 | 🞏 | 🞏 | 🞏 | 🞏 |
| how often have you felt difficulties were piling up so high that you could not overcome them? | 🞏 | 🞏 | 🞏 | 🞏 | 🞏 |

11. Do you suffer or have you suffered from any mental illness? Yes/No

12. Did you have any contact with someone in your family who suffered from mental illness? Yes/No

13. Stigma in mental illness (The stigma of mental illness is assessed using the AQ-9 Attribution questionnaire in its Portuguese version, revised and abbreviated.) (**)

José is a 23-year-old single medical student who is studying for National Test of Access. Recently, he sleeps 3 hours a night, cries constantly, feels exhausted and has no motivation to study. He isolated himself from his friends and showed some aggression towards his parents. He has been hospitalized twice for episodes of self-harm.

|  | No/nothig | | | | Very/completly | | | | |
| --- | --- | --- | --- | --- | --- | --- | --- | --- | --- |
| I would feel pity for José | ➀ | ➁ | ➂ | ➃ | ➄ | ➅ | ➆ | ➇ | ➈ |
| How dangerous would you feel José is? | ➀ | ➁ | ➂ | ➃ | ➄ | ➅ | ➆ | ➇ | ➈ |
| How scared of José would you feel? | ➀ | ➁ | ➂ | ➃ | ➄ | ➅ | ➆ | ➇ | ➈ |
| I would think that it was José’s own fault that he is in the present condition | ➀ | ➁ | ➂ | ➃ | ➄ | ➅ | ➆ | ➇ | ➈ |
| I think it would be best for José’s community if he were put away in a psychiatric hospital | ➀ | ➁ | ➂ | ➃ | ➄ | ➅ | ➆ | ➇ | ➈ |
| How angry would you feel at José? | ➀ | ➁ | ➂ | ➃ | ➄ | ➅ | ➆ | ➇ | ➈ |
| How likely is it that you would help José? | ➀ | ➁ | ➂ | ➃ | ➄ | ➅ | ➆ | ➇ | ➈ |
| I would try to stay away from José. | ➀ | ➁ | ➂ | ➃ | ➄ | ➅ | ➆ | ➇ | ➈ |
| How much do you agree that Harry should be forced into treatment with his doctor even if he does not want to? | ➀ | ➁ | ➂ | ➃ | ➄ | ➅ | ➆ | ➇ | ➈ |

(*) Ribeiro JP, Marques T. A avaliação do stresse: a propósito de um estudo de adaptação da escala de percepção de stresse. *Psicol Saúde Doenças*. 2009;10(2):237-248.

(**) Corrigan P, Markowitz FE, Watson A, Rowan D, Kubiak MA. An attribution model of public discrimination towards persons with mental illness. *J Health Soc Behav*. 2003;44(2):162-179. doi:10.2307/1519806; Corrigan P. *A TOOLKIT for Evaluating Programs Meant to Erase the Stigma of Mental Illness*.; 2008.
